# Bio-Inspired Aquaporinz Containing Double-Skinned Forward Osmosis Membrane Synthesized through Layer-by-Layer Assembly

**DOI:** 10.3390/membranes5030369

**Published:** 2015-08-10

**Authors:** Shuzheng Wang, Jin Cai, Wande Ding, Zhinan Xu, Zhining Wang

**Affiliations:** 1 Key Laboratory of Marine Chemistry Theory and Technology, Ministry of Education, Ocean University of China, Qingdao 266100, China; E-Mails: hdhxeb@163.com (S.W.); dingwande@163.com (W.D.); 2 Institute of Biological Engineering, College of Chemical and Biological Engineering, Zhejiang University, Hangzhou 310027, China; E-Mails: caij@zju.edu.cn (J.C.); znxu@zju.edu.cn (Z.X.)

**Keywords:** Aquaporinz, supported lipid bilayer, layer-by-layer, double-skinned, biomimetic membrane, forward osmosis

## Abstract

We demonstrated a novel AquaporinZ (AqpZ)-incorporated double-skinned forward osmosis (FO) membrane by layer-by-layer (LbL) assembly strategy. Positively charged poly(ethyleneimine) (PEI) and negatively charged poly(sodium 4-styrenesulfonate) (PSS) were alternately deposited on both the top and bottom surfaces of a hydrolyzed polyacrylonitrile (H-PAN) substrate. Subsequently, an AqpZ-embedded 1,2-dioleloyl-sn-glycero-3-phosphocholine (DOPC)/1,2-dioleoyl-3-trimethylammonium- propane (chloride salt) (DOTAP) supported lipid bilayer (SLB) was formed on PSS-terminated (T-PSS) membrane via vesicle rupture method. The morphology and structure of the biomimetic membranes were characterized by *in situ* atomic force microscopy (AFM), scanning electron microscope (SEM), Fourier transform infrared spectrometer using the attenuated total reflection technique (ATR-FTIR), and contact angle. Moreover, the FO performance of the resultant membrane was measured by using 2 M MgCl_2_ solution as draw solution and deionized (DI) water as feed solution, respectively. The membrane with a protein-to-lipid weight ratio (P/L) of 1/50 exhibits 13.2 L/m^2^h water flux and 3.2 g/m^2^h reversed flux by using FO mode, as well as 15.6 L/m^2^h water flux and 3.4 L/m^2^h reversed flux for PRO mode (the draw solution is placed against the active layer). It was also shown that the SLB layer of the double-skinned FO membrane can increase the surface hydrophilicity and reduce the surface roughness, which leads to an improved anti-fouling performance against humic acid foulant. The current work introduced a new method of fabricating high performance biomimetic FO membrane by combining AqpZ and a double-skinned structure based on LbL assembly.

## 1. Introduction

Aquaporins (AQPs) are a kind of transmembrane water channel protein that selectively allow the transport of water molecules because of their narrow channels with a dimensional structure and distinct charge characteristics [1]. It has been reported that the osmotic permeability of a single AQP is between 6 × 10^−14^ to 24 × 10^−14^ m^3^·s^−1^ [2]. AQP-containing biomimetic membranes hold great potential in water purification and seawater desalination due to the excellent permeability and the selectivity of AQP. AqpZ, the simplest member of the AQP family, is of particular interest for water treatment and desalination because of its lack of N-linked glycan, its resistance to proteolysis, and its high osmotic water permeability [3].

To date, several methods have been applied to fabricate AQP-containing biomimetic membranes, most of which are based on depositing AQP-incorporated vesicles onto porous substrates. Many efforts have been made to improve the performance of the AQP-based biomimetic membranes, including direct deposition of vesicles onto the substrates, pressure-assisted vesicle fusion, electrostatic force-induced vesicle adsorption, magnetic-enhanced vesicle deposition, and chemical crosslinking between functionalized lipid and the substrate [4,5,6,7]. Wang *et al.* reported on an AqpZ-embedded supported lipid bilayer (SLB) by using an electrostatic layer-by-layer (LbL) deposition method to prepare a robust biomimetic membrane, which demonstrated an excellent nanofiltration (NF) performance [4]. Li *et al.* fabricated SLB biomimetic membranes by spin-coating positively charged proteoliposomes on a modified NF-270 membrane via vesicle fusion [5]. Sun *et al.* encapsulated magnetic nanoparticles into the vesicles and utilized magnetic force to enhance the adsorption amount of AqpZ-incorporated vesicles on the polyelectrolyte film [6]. Wang *et al.* reported a method of synthesizing a FO biomimetic membrane [7]. In their process, the UV-crosslinked AqpZ-ABA block copolymer proteopolymersomes were immobilized on the membrane support, followed by stabilization through a layer-by-layer polydopamine-histidine coating process [7].

Forward osmosis (FO) utilizes an osmotic pressure gradient across the semipermeable membrane to separate clean water from feed solution (FS) [8]. Advantages of FO over pressure-driven membrane processes include lower energy consumption, less fouling propensity, and easy cleaning [8,9]. There are more and more fields where FO can be applied, such as seawater desalination, wastewater treatment, FO-based membrane bioreactors, food processing, and power generation, *etc.* [9,10,11]. However, due to the asymmetric structure of the traditional FO membranes, the phenomenon called “internal concentration polarization” (ICP) is created severely, and it can lower the effective osmotic driving force for water transport and limit the water flux of the FO process to a large extent. When the feed solution is placed against the active layer (FO mode), dilutive ICP occurs within the membrane support layer as water permeates across the membrane from FS to draw solution (DS). On the contrary, concentrative ICP occurs as the solute in the feed solution accumulates within the membrane support layer, when the draw solution is placed against the active layer (PRO mode) [12,13]. In the FO process, ICP usually causes a severe decline in water flux in the FO mode [14,15]. However, foulants can hardly enter the pores of the porous support. For PRO mode, ICP is drastically reduced, but foulants tend to go into the porous support easily [16]. In order to make full use of the advantages of the two different modes, double-skinned FO membranes have been designed and fabricated [17,18]. Compared with traditional single-skinned FO membranes, a dense rejection skin is formed on the bottom surface of the membrane. This additional skin can prevent solute reverse diffusion and reduce ICP effectively [19].

**Figure 1:**
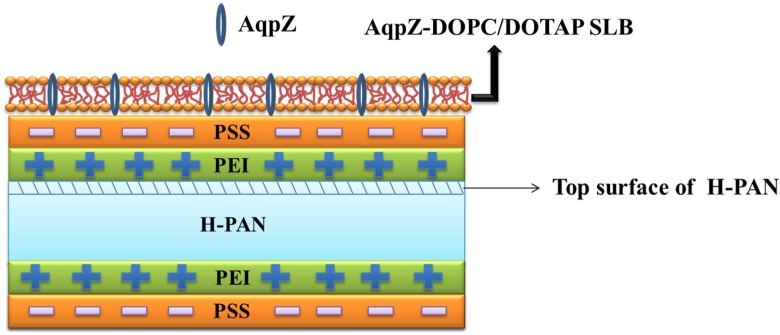
Schematic diagram of the formation procedures for AqpZ-incorporated double-skinned FO membrane.

In our work, we combined AqpZ with double-skinned FO membranes to further improve the performances of the biomimetic FO membranes. The electrostatic LbL deposition method, which is based on the alternating adsorption of oppositely charged components [20,21], has been demonstrated to form a robust biomimetic membrane [4]. Thus, we used this method to fabricate T-PSS membranes on the basis of porous substrates. AqpZ-embedded SLB was formed via vesicle rupture to obtain the bio-inspired AqpZ containing the double-skinned FO membrane. As shown in Figure 1, on the top surface of a hydrolyzed polyacrylonitrile (H-PAN), poly(ethyleneimine) (PEI) was deposited to form the polycation layer, then polystyrene sulfonate (PSS) was deposited to form the polyanion layer. The process was repeated on the bottom surface of H-PAN. The positively charged 1,2-dioleloyl-sn-glycero-3-phosphocholine (DOPC) and 1,2-dioleoyl-3-trimethylammo-nium-propane (chloride salt) (DOTAP) mixture proteoliposomes were deposited onto the top surface of the LbL membrane to form a SLB. In the current work, we prepared three kinds of membranes: PEI-terminated double-skinned (T-PEI) FO membranes, PSS-terminated double-skinned (T-PSS) FO membranes, and AqpZ-incorporated double-skinned (T-AqpZ) FO membranes. FO performances and fouling tests of AqpZ-incorporated double-skinned FO membranes were investigated and compared with the single-skinned FO membranes.

## 2. Experimental

### 2.1. Materials and Chemicals

Polyacrylonitrile (PAN, Mw ~50,000) was kindly supplied by the Shanghai Jingshan Petrochemical Company. *N,N*-dimethylformamide (DMF) and lithium chloride were purchased from Sinopharm (Shanghai, China). Polyethyleneimine (PEI, Mw ~750,000) and poly(sodium 4-styrene-sulfonate) (PSS, Mw ~70,000), 1-n-octyl-β-d-glucopyranoside (OG, purity > 99%) and glycerol (purity > 99%) were purchased from Sigma-Aldrich (St. Louis, MO, USA). 1,2-dioleloyl-sn-glycero-3-phosphocholine (DOPC, purity > 99%) and 1,2-dioleoyl-3-trimethylammo-nium-propane (chloride salt) (DOTAP) were obtained from Avanti Polar Lipids (USA). AqpZ was expressed and purified using previously described procedures [22]. Deionized water (Millipore) with a resistivity of 18.2 MΩ·cm was used to prepare buffer solutions and for FO measurement. Buffer solution used through the experiment was phosphate buffer solution (pH = 7.5) prepared with 10 mM KH_2_PO_4_, 10 mM K_2_HPO_4_ 3H_2_O, and 150 mM NaCl.

### 2.2. Preparation of Liposomes and Proteoliposomes

Liposome samples were prepared by the film rehydration method as previously described [23]. Briefly, a N_2_ stream was used to evaporate chloroform from a chloroform solution of DOPC/DOTAP mixture with a molar ratio of DOPC: DOTAP = 4:1 to obtain a lipid film. The dried lipid film was left standing overnight in a vacuum to remove the residual organic solvent, and then rehydrated in phosphate buffer through vortexing, followed by five freeze-thaw cycles. The final solution was extruded 11 times with 100 nm polycarbonate membranes through an extruder system (Avanti Polar Lipids, Alabaster, AL, USA) to get the uniform small unilamellar vesicles with a final concentration of 0.1 mg/mL.

A certain amount of AqpZ solution was added into 0.1 mg/mL liposome solution containing 1 wt % OG to prepared samples with different protein-to-lipid ratios (P/L = 1/200, 1/100, 1/50). The solution was dialyzed in a 6–8 KDa molecular weight cut-off (MWCO) dialysis bag (Spectra Por) against 1 L phosphate buffer for three days at room temperature. The buffer was refreshed every day. After dialysis, the proteoliposome solution was extruded through a polycarbonate membrane with a mean pore size of 100 nm as stated above for further use.

### 2.3. Preparation of PAN Substrates

PAN substrate was fabricated by using the phase inversion method. The PAN casting solution consisted of 18 wt % PAN and 2 wt % LiCl in DMF. The solution was stirred for 24 h at 60 °C to obtain a homogeneous solution and then cooled to room temperature for 24 h to remove the residual bubbles. A casting knife was used to cast the homogeneous solution on a clean glass plate, and then the glass plate was immediately immersed into a distilled water coagulation bath to form PAN substrates. Subsequently, the PAN substrates were soaked in a 1.5 M NaOH solution at 45 °C for 1.5 h and then rinsed by DI water until the pH of the rinse water became neutral [24]. The alkali solution-treated PAN membrane was denoted as H-PAN.

### 2.4. Fabrication of T-PSS Membrane

The LbL membrane was prepared by immersing the H-PAN substrate in 1 wt % PEI and 1 wt % PSS solution alternatively. Each deposition step lasted 30 min followed by a rinse of DI water for 5 min to remove the redundant polyelectrolytes. The procedure above could be repeated so that multilayer polyelectrolyte membranes with a specific bilayer number were fabricated. In our work, we adopted one PEI/PSS layer for the top rejection skin and then a similar method was used for the preparation of the bottom rejection layer. In that way, T-PSS membranes were fabricated.

### 2.5. AqpZ-Incorporated SLB Formation on T-PSS Membrane

The prepared liposome or proteoliposome solutions were spread on the top layer of the T-PSS membrane and incubated at room temperature for 4 h. Then the biomimetic membrane was rinsed by DI water for 5 min to remove the residual vesicles.

### 2.6. Vesicle Characterizations

Zetasizer (NanoZS 90, Malvern Instruments Limited, Worcestershire, UK) was used to characterize the size and zeta potential of liposomes and proteoliposomes. A 633 nm laser source, with a fixed detector angle of 90°, was used in the dynamic mode. We used a stopped-flow apparatus (Applied Photophysics Ltd., Surrey, UK), with light source at emission wavelength of 577 nm, to characterize the permeability of the liposomes and proteoliposomes. Samples were rapidly mixed with a high-osmotic solution (0.5 mol/L sucrose). The osmolarity difference (*∆*_osm_ = 290 mosm/L, Osmomat 030, Gonotec, Germany) impelled the water effluent from the vesicles. By fitting the data to an exponential increase equation, the initial rate of vesicle shrinkage (*k*) was determined. Thereafter, the osmotic water permeability (*P_f_*) was calculated by using the following equation:

*P_f_* = *k* / ((*S*/*V*_0_) × *V*_W_ × Δ_osm_) (1)

where *S/V*_0_ is the vesicle’s initial surface-to-volume ratio, *V*_W_ is the molar volume of water (0.018 L/mol), and *∆*_osm_ is the osmolarity difference after mixing. All of the measurements were carried out at 25 ± 0.1 °C.

### 2.7. Membrane Characterizations

The functionalized groups of the resultant membrane were detected by the Fourier transform infrared (FTIR) spectrometer (Tensor 27, Bruker, Germany), using the attenuated total reflection (ATR) technique.

The hydrophilicity of the prepared membrane was characterized by an automatic contact angle meter (DSA100, Kruss, Germany). Then 0.5 mL DI water was dropped onto the membrane surface using a microsyringe under ambient conditions and contact angle was measured until no further change. At least six random locations were measured for each sample in order to minimize the experimental error and then obtain the average value. Both the top surfaces and bottom surfaces of the double-skinned LbL FO membranes were measured.

The surface morphology and roughness of the membranes were investigated via atomic force microscopy (AFM, Nanoscope V MultiMode, Veeco, Plainview, NY, USA). Tapping mode measurements in liquid were performed by using NP-S cantilevers (short lever, nominal spring constant 0.06 N/m). Surface roughness values were obtained from AFM images with the help of the instrument software.

The morphology and structure of original and modified membranes were analyzed by scanning electron microscope (SEM, S-4800, Hitachi, Japan). Dry membrane samples were frozen in liquid nitrogen and subsequently cracked in order to obtain the cross sections.

### 2.8. FO Performance

FO performances of the fabricated membranes were conducted in a lab-scale cross-flow FO filtration unit with an effective membrane area of 36.0 cm^2^. The linear velocity was 6.4 cm/s. We denoted the AqpZ-embedded SLB active layer facing FS as the FO mode. Accordingly, the active layer facing DS was denoted as pressure retarded osmosis (PRO) mode. The FO performances of double-skinned membranes were evaluated under both FO and PRO mode. As a control, single-skinned membranes, which were prepared by depositing only the top layer of H-PAN with polyelectrolytes and AqpZ-embedded SLB, were also measured.

Then, 2 mol/L MgCl_2_ solution was used as DS and DI water was used as FS, respectively. The water flux was calculated by measuring the weight change of DS every 2 min. The solute reverse flux of the membrane was calculated from the conductivity change.

The water flux (*J*_v_ in L/m^2^·h) and solute reverse flux (*J*_s_ in g/m^2^·h) were calculated by the following equations [25,26]:

(2) $$J_{\text{v}}=\frac{∆V}{A_{\text{m}}\times ∆t}$$

where Δ*V* is the volume changes of DI water (density is 1000 g/L); *A*_m_ is the effective membrane area; Δ*t* is the measuring time interval.

(3) $$J_{\text{s}}=\frac{C_{t}V_{t}-C_{0}V_{0}}{A_{\text{m}}\times ∆t}$$

where *V*_0_ and *V_t_* are the initial and final volumes of feed solution, respectively; *C*_0_ and *C_t_* are the initial and final solute concentrations of feed solution, respectively.

The anti-fouling tests of double-skinned membranes were performed in the FO mode. Prior to the fouling test, the FO membrane was first tested using 2 mol/L MgCl_2_ DS and DI water FS for 1 h to obtain the initial water flux. Anti-fouling tests were performed with 5 mg/L humic acid (HA) as the FS for 1 h. Subsequently, both the FS and DS were changed into DI water to rinse the membrane for 0.5 h, which was the cleaning process. Finally, 2 mol/L MgCl_2_ DS and DI water FS were used for 1 h to measure the recovered water flux. The fouling and cleaning cycles were performed three times, alternatively.

## 3. Results and Discussion

### 3.1. Characteristics of Liposomes and Proteoliposomes

Table 1 summarizes the size and zeta potential of DOPC, DOPC/DOTAP liposomes, and proteoliposomes at different P/L (w/w) ratios. The vesicles exhibited a narrow size range from 114 to 122 nm and their polydispersity index (PDI) was consistently smaller than 0.2, indicating a narrow size distribution of the vesicles [27]. Moreover, proteoliposomes showed decreased zeta potential after incorporation of AqpZ because the incorporated proteins were negatively charged in phosphate buffer saline PBS (pH = 7.5) [28,29]. Apparently, the incorporation of AqpZ had a major influence on the physicochemical properties of the vesicles.

**Table 1:** Characterization of DOPC, DOPC/DOTAP liposomes, and proteoliposomes.

Samples	Hydrodynamic Diameter of Vesicles (nm)	Poly-Dispersion Index (PDI)	Zeta Potential (mV)	*k* (s^−1^)	*Pf* (μm·s^−1^)
DOPC liposomes	122.9	0.175	−0.99	52.10	204.09
DOPC/DOTAP liposomes	114.0	0.119	10.34	71.91	261.29
DOPC/DOTAP proteoliposomes (P/L = 1/200)	115.6	0.085	7.16	688.76	2537.78
DOPC/DOTAP proteoliposomes (P/L = 1/100)	116.1	0.096	6.08	749.61	2773.94
DOPC/DOTAP proteoliposomes (P/L = 1/50)	116.8	0.108	4.96	839.97	3127.05

Table 1 also summarized the water permeability data. The permeability of AqpZ-incorporated proteoliposomes was measured by the stopped-flow apparatus. Here, proteoliposomes showed a much higher shrinkage rate (*k*) than liposomes, which could be attributed to the higher water permeation of AqpZ. Moreover, the permeability of proteoliposomes increased as the P/L ratio increased because more incorporated AqpZ enhanced the permeability of the vesicles. Based on the data of the proteoliposomes with the protein-to-lipid molar ratio of 1:50, the permeability of each AqpZ was calculated to be 32.4 × 10^−14^ cm^3^·s^−1^, which was comparable to the previously reported results [30,31].

### 3.2. ATR-FTIR Characterization

**Figure 2:**
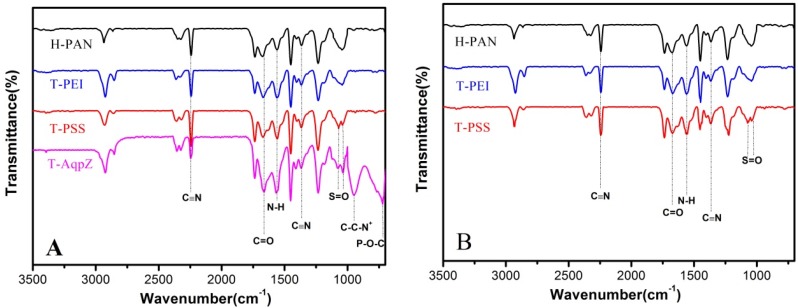
ATR-FTIR spectra of various membranes. (**A**) Top surfaces of membranes; (**B**) Bottom surfaces of membranes.

ATR-FTIR analyses were performed to verify that the AqpZ-incorporated double-skinned FO membranes were successfully fabricated. As shown in Figure 2A, the peak at 2245 cm^−1^ was due to stretching vibration of the C≡N moiety of the PAN substrate. After being treated by 1.5 M NaOH solution, most of the CN groups convert to COO^−^ groups as previously reported [4]. As a result, the H-PAN membrane exhibited a peak at approximately 1677 cm^−1^, representing the C=O bond in COO^−^ groups. After PEI deposition, the intensity of the N–H bend and C–N stretch of the CONH group increased at around 1553 cm^−1^, which suggested that the nucleophilic amine groups were formed on the membrane. For the T-PSS membrane, two new peaks appeared at 1068 cm^−1^ and 1042 cm^−1^, which corresponded to the S=O bond stretching vibration. When AqpZ-incorporated DOPC/DOTAP SLB was formed on the polyelectrolyte membrane (the membrane we denoted as T-AqpZ), the characteristic peak attributed to C–C–N^+^ groups appeared at around 947 cm^−1^. As well, the peak at 715 cm^−1^ was attributed to the vibration of P–O–C groups. The spectra of the membrane bottom surfaces with different outermost layers were shown in Figure 2B. The characteristic peaks belonging to H-PAN, PEI, and PSS could be found, which demonstrated that the AqpZ-incorporated FO membrane with a double-skinned structure was successfully fabricated.

### 3.3. Hydrophilicity of Membranes

**Figure 3:**
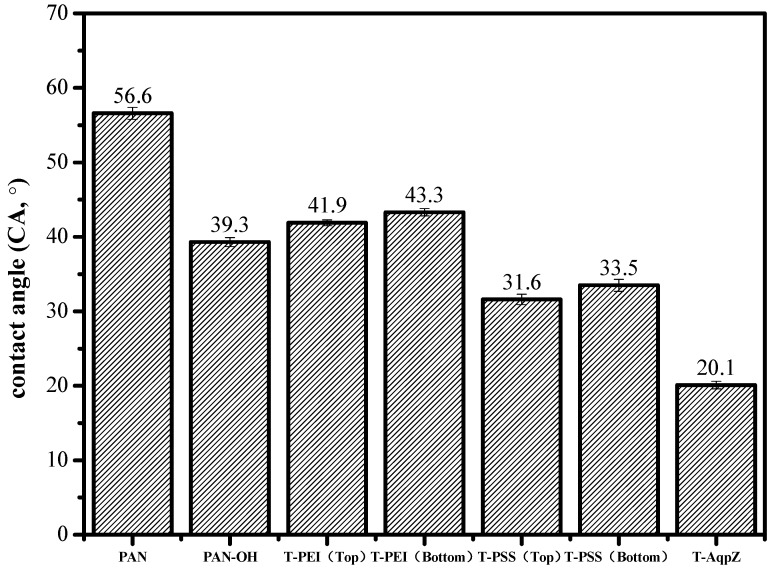
Contact angles of PAN, H-PAN, T-PEI (top surface), T-PEI (bottom surface), T-PSS (top surface), T-PSS (bottom surface), and T-AqpZ membrane.

The hydrophilicity of the membrane surface is usually evaluated by the water contact angle. In general, a higher contact angle of the membrane surface indicates higher hydrophobicity [32]. The water contact angles of the FO membranes are illustrated in Figure 3. The water contact angle of PAN was 56.6°. After deposition of PEI/PSS, the water contact angle dropped to 31.6°. This can be understood by the fact that acidic COO^−^ groups and some amine groups on the surface of the T-PSS membrane have a strong interaction with water molecules. Finally, after covering with proteoliposomes, the water contact angle made a further decrease to 20.1°. This can be understood by the fact that the SLB with high hydrophilicity covered the surface of the membrane and greatly improved the surface hydrophilicity [33].

### 3.4. Morphologies of the FO Membrane

**Figure 4:**
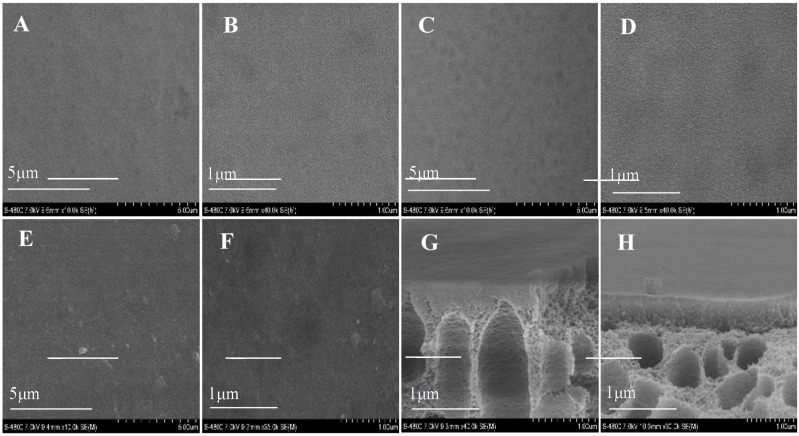
SEM images of various membranes. (**A**,**B**) top surface of T-PSS membrane; (**C**,**D**) bottom surface of T-PSS membrane; (**E**,**F**) top surface of T-AqpZ membrane; (**G**) cross-section of top surface of T-PSS membrane; (**H**) cross-section of bottom surface of T-PSS membrane.

The SEM images of fabricated membranes are shown in Figure 4. After the deposition of AqpZ*-*embedded SLB, the gaps in the top surface were filled so that the membrane appeared to be smoother and less defective. As for the cross-section images, finger-like pores were formed in the asymmetric membranes [34]. Both the top surface and the bottom surface had a dense selective layer and this layer on the bottom surface was looser than that on the top surface, which was attributed to the difference of roughness and pore size of the top surface and the bottom surface. This was due to the fact that the large pores on the bottom surface led to an uneven deposition of polyelectrolytes and proteoliposomes. The cross-section images proved the fabrication of the double-skinned membrane structure [17].

### 3.5. AFM Characterization

**Figure 5:**
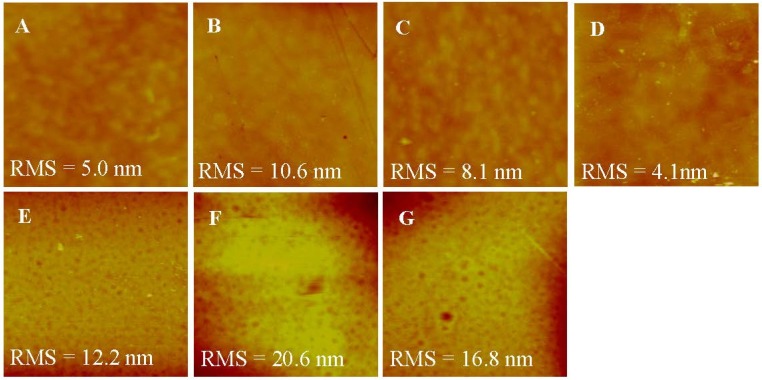
AFM images (scan size 2 × 2 μm^2^，z-scale = 100 nm) of the various membranes. (**A**) Top surface of H-PAN; (**B**) Top surface of T-PEI; (**C**) Top surface of T-PSS; (**D**) Top surface of T-AqpZ; (**E**) Bottom surface of H-PAN; (**F**) Bottom surface of T-PEI; (**G**) Bottom surface of T-PSS.

AFM was used to characterize the roughness of various membranes. As shown in Figure 5, top surfaces of the membranes were flatter than bottom surfaces, correlating well with the SEM images. The roughness of the top surface ranged from 4.131 to 10.638 mm, which was much smaller than the roughness of the bottom surface, ranging from 12.215 to 20.641 mm. This might be induced by more large pores existing at the bottom surface. After LbL deposition, the roughness of both the top and the bottom surface of membranes became higher than the H-PAN substrate because of the random adsorption of polyelectrolytes on the membranes [35,36]. Moreover, the PSS layer led to the decrease of roughness due to the shorter and more inflexible hydrocarbon chains of PSS than PEI. When the SLB was formed on the T-PSS membrane, the roughness decreased accordingly, and was even lower than the H-PAN substrate [35]. This can be understood by the fact that AqpZ-embedded SLB filled up the gaps on the substrates, which in turn proved the successful formation of AqpZ-embedded SLB.

### 3.6. FO Performances of Double-Skinned Membranes

**Figure 6:**
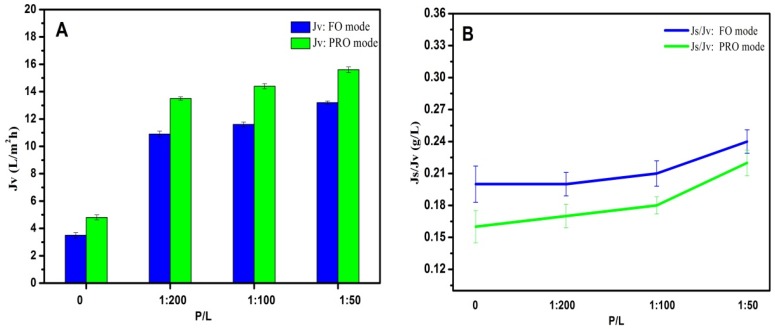
FO performances of double-skinned membranes by using 2 mol/L MgCl_2_ as the DS and DI water as the FS. (**A**) *J*_v_ of double-skinned membranes; (**B**) *J*_s_/*J*_v_ of double-skinned membranes.

The FO performance of AqpZ-incorporated double-skinned FO membranes was evaluated by using a 2M MgCl_2_ solution as the DS and DI water as the FS. Both the FO and PRO modes (the draw solution is placed against the active layer) were measured and shown in Figure 6. The *J*_v_ of AqpZ-absent SLB-covered double-skinned FO membrane was 3.5–4.8 L/m^2^·h and the *J*_s_/*J*_v_ was about 0.2 g/L. The low water flux could be explained by the fact that the lipid bilayer was impermeable to water. However, due to the existence of defects on the SLB, the membrane exhibited a low water flux and reversed salt solution flux [5]. After the embedding of AqpZ with different protein-to-lipid ratios (P/L from 1/200 to 1/50), *J*_v_ increased from 13.7 to 15.6 L/m^2^·h by using the PRO mode, while the *J*_s_/*J*_v_ slightly increased from 0.21 to 0.24 g/L. This can be understood by considering the fact that plenty of water channels were provided by AqpZ for water molecules to pass through the biomimetic membranes. However, the zeta potential of proteoliposomes decreased with the increase of the P/L ratio (as shown in Table 1). Therefore, the interaction between the polyelectrolyte layer and the SLB layer weakened, and then more defects appeared and induced the increase of reversed solute flux. A similar phenomenon was also observed by Wang *et al.* [37]. It was also worth noting that the membranes exhibited higher *J*_v_ and lower *J*_s_/*J*_v_ at the PRO mode than at the FO mode. This was mainly because of the denser top layer at the PRO mode, which prevented more draw solutes from entering into the membrane and reduced the ICP level as a result [17].

### 3.7. FO Performances of Single-Skinned and Double-Skinned FO Membranes

**Figure 7:**
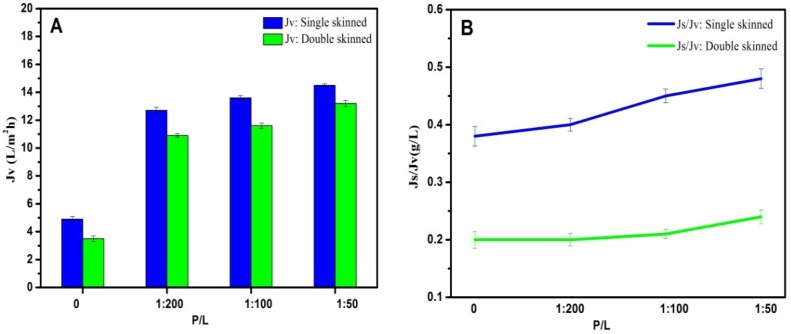
FO performances of AqpZ-incorporated single-skinned and double-skinned FO membranes in the FO mode. (**A**) *J*_v_ of AqpZ-incorporated single-skinned and double-skinned FO membranes; (**B**) *J*_s_/*J*_v_ of AqpZ-incorporated single-skinned and double-skinned FO membranes.

As shown in Figure 7, AqpZ-incorporated single-skinned FO membranes were also measured for comparison. Both the double-skinned membranes and single-skinned membranes were performed in the FO mode, where the active layer faced the FS. In the FO mode, dilutive ICP occurs within the membrane support layer as water permeates across the membrane from the FS to the draw solution, which usually causes a severe decline in water flux, so the flux experiments were performed in the FO mode to confirm that the double-skinned FO membrane could reduce the ICP level. The single-skinned FO membranes incorporated with AqpZ (P/L = 1/50) had the highest *J*_v_ at 14.5 L/m^2^·h. The reason for this result could be related to two aspects. Firstly, single-skinned FO membranes possessed less resistance than double-skinned FO membranes because of the lack of a secondary selective layer. Secondly, membranes with P/L = 1/50 provided more water channels than membranes with P/L = 1/100 and P/L = 1/200, which allowed more water molecules to pass through the membrane. Although single-skinned membranes showed higher *J*_v_, the double-skinned structure significantly reduced *J*_s_/*J*_v_, indicating the higher solute rejection of the double-skinned FO membranes. Both kinds of membranes showed excellent abilities of water separation compared to membranes without AqpZ (P/L = 0), which confirmed the fact that AqpZ was beneficial to FO biomimetic membranes.

### 3.8. FO Fouling Tests

**Figure 8:**
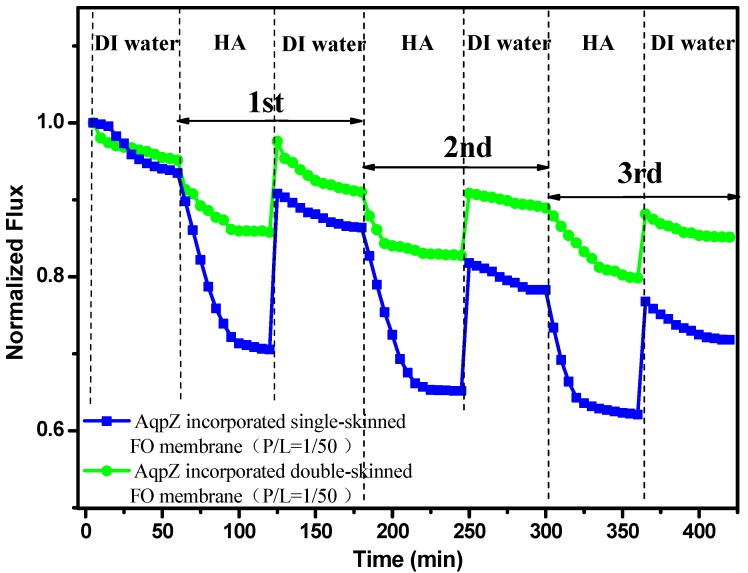
Normalized fluxes of AqpZ-incorporated single-skinned and double-skinned FO membranes during three cycles of humic acid (HA) solution and DI water FO process.

The FO fouling tests were performed with AqpZ (P/L = 1/50)-incorporated membranes in the PRO mode. In the PRO mode, foulants could go into the porous support easily. Therefore, the PRO mode was used to test the anti-fouling capacity of the double-skinned FO membrane. Figure 8 shows the comparison of double-skinned FO membranes and single-skinned FO membranes with normalized fluxes. Before fouling, the initial water fluxes of both membranes were measured by using 2M MgCl_2_ as the DS and DI water as the FS for 1 h. The water flux slightly dropped because of the decrease of the driven pressure induced by the dilutive ICP [13]. Remarkably, the double-skinned membrane had more advantages in the anti-fouling test. The single-skinned membrane suffereda severe decline in water flux (decreased to ~70.5%) within a few minutes after the fouling test. Similar results were reported for several types of single-skinned FO membranes [9,17]. This can be understood by the fact that HA in FS could easily enter into the pores of the PAN substrates and cause increased membrane resistance and reduced water molecule transport, which in turn causes severe flux loss [17]. However, the water flux of double-skinned membranes just decreased slightly (decreased to ~85.5%), which demonstrated that the double-skinned FO membrane could effectively mitigate fouling. This may be caused by the fact that the added secondary selective layer was dense enough to prevent the foulant from clogging the water molecule channels in the membrane [17]. Moreover, the double-skinned FO membranes exhibited higher normalized recovery flux at 85.1%, whereas, the normalized recovery flux of single-skinned FO membranes was 71.8% after three cycles of fouling and cleaning. It indicated that the double-skinned FO membranes possessed huge potential in anti-fouling capacity. Our work provided a novel structure of AqpZ-incorporated FO membranes, which enhanced the anti-fouling stability and offers a clear advantage for FO applications.

## 4. Conclusions

We successfully fabricated and characterized the AqpZ-incorporated double-skinned FO membranes based on layer-by-layer assembly in the current work. To the best knowledge of the authors, this is the first study reporting the combination of AqpZ and double-skinned FO membranes. The addition of AqpZ has made a great contribution to the water flux and salt rejection. The changes were obvious in the water flux. The double-skinned FO membranes possessed a relatively loose secondary selective layer, which decreased ICP and ensured the water flux. Through fouling tests, the double-skinned FO membranes demonstrated a much better anti-fouling capacity compared to the single-skinned FO membranes.
